# Genomic Analysis and Surveillance of the Coronavirus Dominant in Ducks in China

**DOI:** 10.1371/journal.pone.0129256

**Published:** 2015-06-08

**Authors:** Qing-Ye Zhuang, Kai-Cheng Wang, Shuo Liu, Guang-Yu Hou, Wen-Ming Jiang, Su-Chun Wang, Jin-Ping Li, Jian-Min Yu, Ji-Ming Chen

**Affiliations:** China Animal Health and Epidemiology Center, Qingdao, 266032, China; Thomas Jefferson University, UNITED STATES

## Abstract

The genetic diversity, evolution, distribution, and taxonomy of some coronaviruses dominant in birds other than chickens remain enigmatic. In this study we sequenced the genome of a newly identified coronavirus dominant in ducks (DdCoV), and performed a large-scale surveillance of coronaviruses in chickens and ducks using a conserved RT-PCR assay. The viral genome harbors a tandem repeat which is rare in vertebrate RNA viruses. The repeat is homologous to some proteins of various cellular organisms, but its origin remains unknown. Many substitutions, insertions, deletions, and some frameshifts and recombination events have occurred in the genome of the DdCoV, as compared with the coronavirus dominant in chickens (CdCoV). The distances between DdCoV and CdCoV are large enough to separate them into different species within the genus *Gammacoronavirus*. Our surveillance demonstrated that DdCoVs and CdCoVs belong to different lineages and occupy different ecological niches, further supporting that they should be classified into different species. Our surveillance also demonstrated that DdCoVs and CdCoVs are prevalent in live poultry markets in some regions of China. In conclusion, this study shed novel insight into the genetic diversity, evolution, distribution, and taxonomy of the coronaviruses circulating in chickens and ducks.

## Introduction

The severe acute respiratory syndrome (SARS) epidemic in 2003 which resulted in 775 human deaths was caused by SARS coronavirus (CoV) [1–2]. The ongoing Middle East respiratory syndrome (MERS) infections in recent years which have claimed hundreds of human lives were caused by MERS CoV [3]. The discovery of these two previously unknown CoVs and other CoVs related to them in animals has greatly broadened our knowledge about the distribution, diversity, and significance of CoVs in both humans and animals [3–7].

CoVs belong to the subfamily *Coronavirinae* in the family *Coronaviridae*, which covers four genera, namely *Alpha*-, *Beta*-, *Gamma*-, and *Deltacoronavirus* [8]. SARS CoV, MERS CoV, and HKU1 CoV which was mentioned below are members of the genus *Betacoronavirus* [8]. All the CoVs having been detected from domestic fowls belong to the genus *Gammacoronavirus*, while some CoVs detected from wild birds have constituted the genus *Deltacoronavirus* [8–9].

The genus *Gammacoronavirus* is represented by the species of *Avian coronavirus* which includes infectious bronchitis virus (IBV) [8]. Two CoVs isolated from whales have also been placed in the genus *Gammacoronavirus* [8, 10–11]. IBV circulates widely in chickens in the world, and causes acute and highly contagious respiratory diseases in chickens of all ages and diminish egg production in hens [12–13]. Additionally, some CoVs highly homologous to IBV have been detected from turkeys, peafowls, and other birds [13–15].

Some CoVs distinct from IBVs and mainly circulate in ducks, pigeons, or geese have been identified [8, 12, 16–18]. For example, we identified in 2013 a CoV dominant (i.e. mainly circulating) in ducks for the first time using next generation sequencing (NGS) technologies, and found that this CoV represents a potential novel species within the genus *Gammacoronavirus*, as indicated by the sequences of three regions in the viral 1ab gene [12]. However, its taxonomic status remains uncertain because its genome sequence and exact distribution are unknown [8, 12, 16–18].

According to the ninth report of International Committee on Taxonomy of Viruses (ICTV), newly identified CoVs are assigned to a genus based on pair-wise evolutionary distances for the seven *Coronaviridae*-wide conserved domains in replicase polyprotein pp1ab: ADP-ribose-1"-phosphatase (ADRP) in nsp3, nsp5, nsp12, nsp13, nsp14, nsp15, and nsp16 [8]. Viruses that share more than 90% amino acid (aa) sequence identity in these seven conserved replicase domains are considered to belong to the same species. This 90% identity threshold serves as the sole species demarcation criterion [8].

In this study, we sequenced and analyzed the genome of a CoV dominant in ducks, and performed a large-scale surveillance of CoVs circulating in poultry, in order to clarify the taxonomic status of the newly identified CoV, and explore the genetic diversity, evolution, detection, and distribution of coronaviruses circulating in poultry.

## Materials and Methods

### Ethics Statement

This study was conducted according to the animal welfare guidelines of the World Organization for Animal Health [19], and approved by the Animal Welfare Committee of China Animal Health and Epidemiology Center. The feces samples, drinking-water samples and swab samples from poultry farms, backyard flocks and live bird markets, were all collected with permission given by multiple relevant parties, including the Ministry of Agricultural of China, China Animal Health and Epidemiology Center, the relevant veterinary section in the provincial and county government. The feces samples were collected from the fresh feces in the ground. The drinking-water samples were collected from the water trough for a group of birds. Swab samples were collected by gently taking smears from the trachea and cloacae of the domestic fowls and then placed in a transport medium.

### Designation of lineages and viruses

Viruses were assigned into lineages based on phylogenetic analysis, and the coronaviruses dominant in chickens and ducks were designated as chicken-dominant CoV (CdCoV) and duck-dominant CoV (DdCoV), respectively. It should be noted that CdCoV may cover some CoVs isolated from the birds other than chickens, and this is the same to DdCoV. Virus strains were designated in the format of lineage/host/place/number/year (e.g. DdCoV/DK/Guangdong/27/2014), and hosts can be abbreviated as “CK” for chicken and “DK” for duck in the designations.

### Genome sequencing and genome analysis

We isolated DdCoV/DK/Guangdong/27/2014 which is abbreviated as DdCoV/GD/2014 from ducks in 2014. The whole genome of DdCoV/GD/2014 was amplified through RT-PCR with a series of primers (see S1 Table). The primers were designed according to the conserved regions in the genomes of the CoVs in genus *Gammacoronavirus* and the regions having been sequenced through NGS [12]. The RT-PCR reactions were performed in a 50-μl reaction system with incubation at 42°C for 30 min and denaturation at 94°C for 60 s, followed by 30 cycles at 94°C for 30 s, 50–55°C (largely depending on the Tm values of the primers) for 30 s, and 72°C for 1–4 min (depending on the length of the amplicons). RT-PCR products were purified with an agarose gel DNA extraction kit (Sangon, Shanghai, China). The amplicons were purified using an agarose gel DNA extraction kit (Takara, Dalian, China) and ligated into the pEASY-T1 cloning vector (TransGen, Beijing, China). Positive clones were sequenced by the ABI 3730xl DNA Analyzer using the pair of M13 primers from both senses. Sequences were assembled and edited manually to generate the whole genome sequence which was further compared to those of IBVs. The DdCoV genome sequence was annotated manually.

### Sample collection for surveillance

A total of 3583 samples were collected from five provinces (Anhui, Guangdong, Jiangsu, Shanghai and Zhejiang) of China, including 982 swab samples from ducks, 2485 swab samples from chickens, 70 feces samples from ducks, 43 drinking-water samples from ducks, and three drinking-water samples from chickens. These 3583 samples were collected from 28 live poultry markets (LPMs), 14 duck farms, and two backyard flocks in May and June of 2014 mainly for surveillance of avian influenza viruses, Newcastle disease viruses and CoVs circulating in poultry in China. The swab sample was collected through taking smears at both cloacal and oropharyngeal tracts of a bird. The feces sample was collected through taking approximately 0.5 ml wet and fresh feces. The drinking-water sample was collected through taking approximately 3.5 ml drinking-water for a group of birds. The swab samples were stored in 1.5 ml phosphate-buffered saline (PBS, pH 7.2) containing 10% glycerol, and the feces samples were stored in 3.5 ml PBS (pH 7.2) containing 10% glycerol, and the drinking-water samples were stored with 0.4 ml glycerol. The samples were stored at 4°C and detected in three days after collection. The samples were stored at—80°C after detection.

### Detection of swab samples

The swab samples were clarified by centrifugation at 10,000 g for 5 min, and the supernatants were inoculated in 10-day-old specific-pathogen-free (SPF) chicken embryonated eggs via the allantoic sac route. The SPF embryonated eggs were purchased from Shandong Healthtec Laboratory Animal Breeding Company (Jinan, China). The inoculated eggs were further incubated for 2 d, and checked twice each day during the incubation period. The dead ones were picked out and stored in a refrigerator. After the incubation period, the allantoic fluids of live embryos were examined using the routine hemagglutination assay. All the hemagglutination-positive allantoic fluids of live embryos and the allantoic fluids of all dead eggs were investigated by RT-PCR for detection of avian influenza virus and Newcastle disease virus (data not shown). They were also examined further by a conserved RT-PCR assay for detection of CoVs, as described below.

### Detection of feces and drinking-water samples

The feces and drinking-water samples were clarified by centrifugation at 10,000 g for 5 min, and then the supernatants were detected through two ways. One was as the same as the detection for swab samples, and the other was directed directly using the following conserved RT-PCR assay for detection of CoVs.

### Detection of CoVs using a conserved RT-PCR assay

The RNA in the collected feces and drinking-water samples or in the allantoic fluids was extracted using an RNeasy Mini Kit (Qiagen, Hilden, Germany), and amplified with the QIAGEN One Step RT-PCR Kit (Qiagen), using a conserved RT-PCR assay designed by ourselves with the primers 5'-GGTTGGGAYTAYCCYAAGTGTGA-3' (upper) and 5'-GAATCIGCCATAWAAACATTRTT-3' (down). The assay amplifies a 545-nucleotide region in the viral 1ab gene, and we have found that this assay can detect some CoVs circulating humans, pigs, chickens, ducks, geese, and pigeons (the data will be published in another paper). The RT-PCR detection was performed in a 25-μL reaction system with incubation at 42°C for 30 min and denaturation at 94°C for 60 s, followed by 30 cycles at 94°C for 30 s, 50°C for 30 s and 72°C for 1 min. RT-PCR products were purified with an agarose gel DNA extraction kit (Sangon, Shanghai, China), and sequenced directly using the ABI 3730xl DNA Analyzer for the following phylogenetic analysis.

### Phylogenetic analysis

Sequences were aligned using the software MUSCLE [19]. Bayesian Information Criterion (BIC) scores of substitution models and phylogenetic relationships were calculated using the software package MEGA 6.0 [20–21]. Phylogenetic relationships were calculated using the model with the lowest BIC score which is assumed to describe the substitution pattern the best. Gaps were handled by partial deletion and bootstrap values were calculated out of 1000 replicates [20, 22].

### Recombinant analysis

Potential sequence recombination was detected using the software Recombination Detection Program (RDP) v3.8, by the methods of RDP, Geneconv, MaxChi, Bootscan, and Chimaera [23–27]. Only the potential recombination events identified by all these five methods with greater than 99% certainty (99% bootstrap support in the case of Bootscan) were accepted.

### Nucleotide sequence accession numbers

A total of 431 sequences were reported originally in this study including the genomic sequence of DdCoV/GD/2014, the sequences covering a tandem repeat of four DdCoVs, and the sequences of the conserved RT-PCR amplicons detected through the surveillance. Their GenBank accession numbers are KM454473, KP006677–KP006687, KP032640–KP032645, KP032665–KP033040 and KP033043–KP033079. The GenBank accession numbers of the 1ab gene sequences of 11 randomly selected CdCoVs used for sequence identity analysis and recombination analysis are DQ834384, FJ888351, AY641576, NC010800, NC001451, JQ977698, JQ977697, JQ088078, DQ646405, KF574761, and FN430415. The GenBank accession numbers of the previously reported genomic sequences of *Bacillus cereus*, *Streptomyces purpureus*, CdCoV/CK/USA/Beaudette/1937, and CdCoV/TK/Ontario/MG10/2007 are WP019889236, WP016137013, NC001451, and NC010800, respectively. The GenBank accession numbers of the some sequences reported previously by others used for phylogenetic analysis are GU396668–GU396671, GU396674–GU396675, GU396679, GU396681, GU396683, GU396685, GU396687–GU396689.

## Results

### The genomic organization of DdCoV

**Fig 1:**
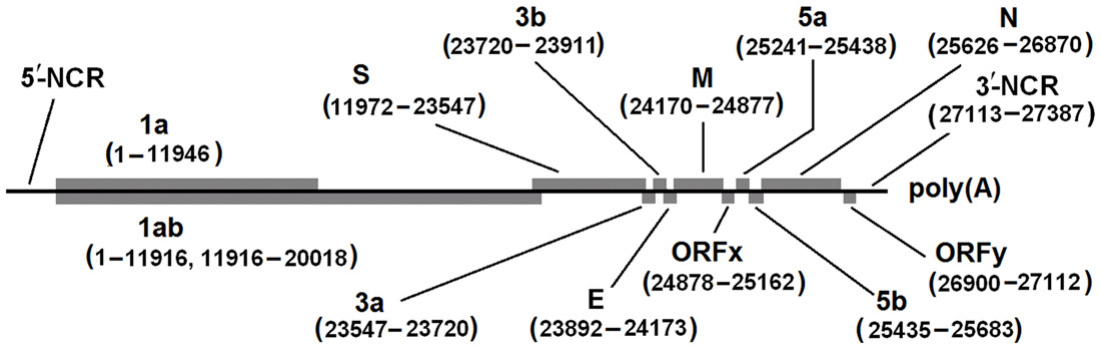
The organization of the genome of DdCoV/GD/2014. ORFs were showed in boxes. Nucleotides were numbered from the first coding nucleotide of the 1a gene after the 5′-NCR.

We sequenced the whole genome of DdCoV/GD/2014 except approximately 170–180 nucleotides (as compared with IBV genomes) at the 5′ non-coding region (NCR) and some adenosines at the 3′ ploy(A) tail. In general, as showed in Fig 1, the genome is approximately 28 kb long with the organization similar to that of CdCoV, and potentially contains 12 open-reading frames (ORFs). Its 5′-most two-thirds are occupied by two overlapping ORFs of the replicase gene, corresponding to polyproteins pp1a and pp1ab. These two polyproteins are potentially cleaved by a set of virus-encoded proteinases into 15 non-structural proteins (nsp2−nsp16) [8]. The 3′-proximal genes code the structural proteins S, E, M, and N. Downstream of the replicase gene and interspersed between the structural protein genes, there are six potential accessory genes (3a, 3b, ORFx, 5a, 5b, and ORFy), the products of which may be key to efficient replication during natural infection [8]. Of these 12 ORFs, 10 have 1–11916 overlapping nucleotides, and there is a −1 ribosomal frameshift within ORF1ab (Fig 1).

### An enigmatic repeat in the genome of DdCoV

**Fig 2:**
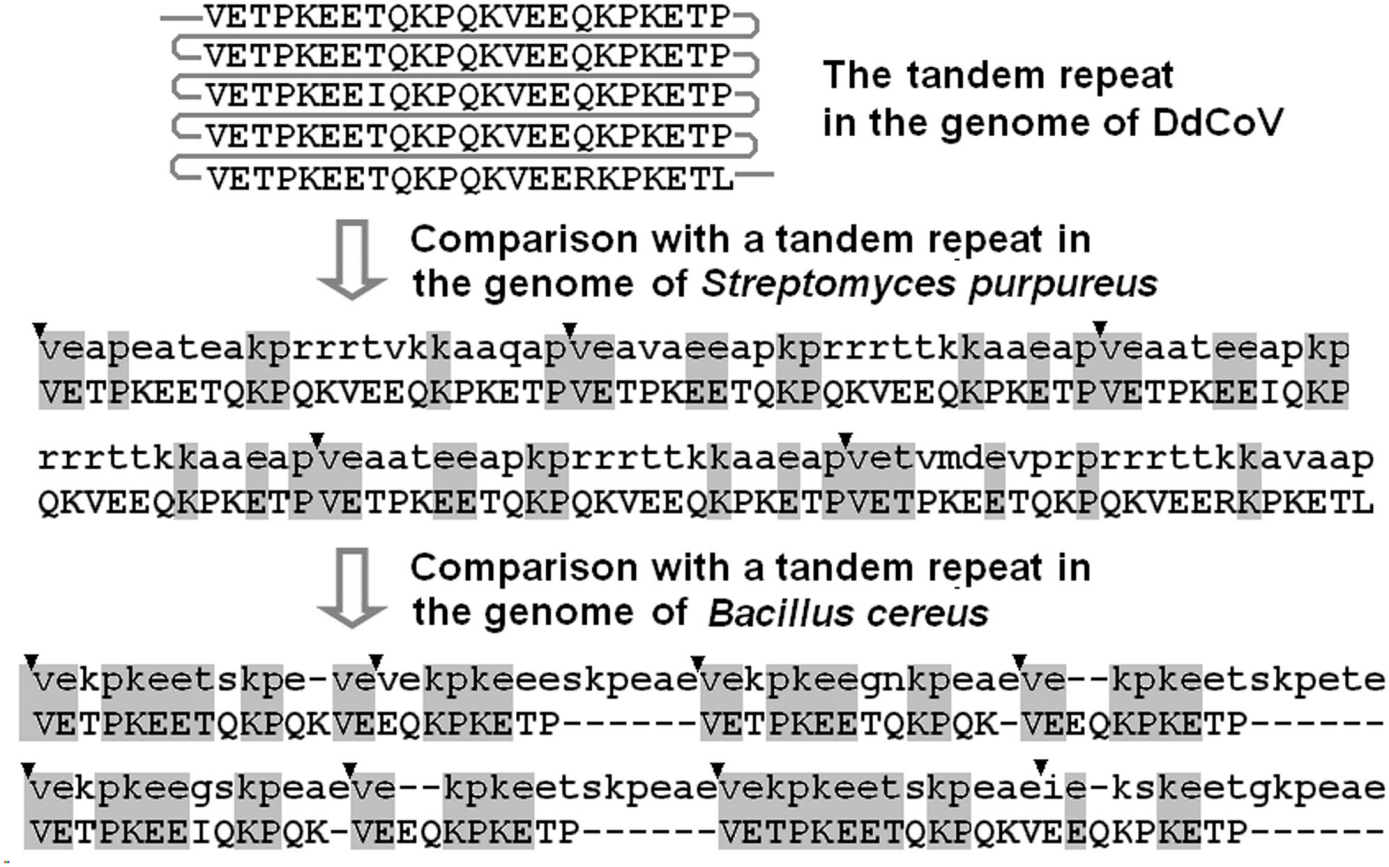
Comparison of the tandem repeats in the genomes of DdCoV (showed in uppercase) and two bacteria (showed in lowercase). The beginning nucleotide of each copy of the bacterial repeats was marked with an arrowhead, and the same residues between the repeats were showed in shadow.

As showed in Fig 2, the nsp3 protein of DdCoV/GD/2014 harbors enigmatically a 5-copy tandem repeat at its papain-like proteinase 1 (PL1^pro^) domain. To further confirm this repeat, we sequenced the region for three times using three pairs of primers (see S1 Table), and obtained the same results. We also sequenced the same region of three other DdCoVs, and found that these three DdCoVs all harbor the 5-copy tandem repeat at the same sites with no nucleotides different from that of DdCoV/GD/2014. The repeat is 115 aa residues long and rich in aa residues of E (*n* = 30), K (*n* = 25), P (*n* = 19), Q (*n* = 14), T (*n* = 14), and V (*n* = 10). These six kinds of aa residues covered 97.4% of the repeat. The repeat is hydrophilic with 56 (48.7%) charged aa residues, and of the net charge of −4.0 at pH 7.0. Only three aa residues and five nucleotide residues are different among the five copies of the tandem repeat.

No hits homologous to the copy of the viral repeat or the whole repeat were found through Megablast in NCBI optimized for search of highly similar nucleotide sequences and Discontiguous Megablast optimized for search of more dissimilar nucleotide sequences, but some hits were identified through Blastn optimized for search of somewhat similar nucleotide sequences, and some hits were identified through Blastp optimized for search of homologous protein sequences [28]. These nucleotide and protein hits were the sequences of various cellular organisms including some bacteria, fungi, animals, and plants, as showed partially in Fig 2. Because the hits may be homologous to the viral repeat by chance, the BLAST data cannot indicate the real origin of the viral repeat.

### Mutations of DdCoV as compared with CdCoVs

As compared with the genome sequences of two randomly selected CdCoVs isolated respectively from chickens and turkeys, CdCoV/CK/USA/Beaudette/1937 and CdCoV/TK/Ontario/MG10/2007, thousands of substitutions and dozens of indels occurred in the genome of DdCoV/GD/2014, and quite a few indels resulted in frameshifts. For example, as compared with the two CdCoVs, >4000 substitutions and >30 indels occurred in the 1ab gene of DdCoV/GD/2014, and >10 of these indels resulted in frameshifts (S1 Fig). Except the one near the end of the ORF, these frameshifts all occurred in pairs with 5–25 nucleotide intervals, and this can minimize the changes in the aa sequences caused by the frameshifts.

### Analysis of the sequences of seven conserved domains of DdCoVs and CdCoVs

The identity between three DdCoVs reported herein including DdCoV/GD/2014 and 11 randomly selected CdCoVs (nine from chickens, one from turkeys, and one from peafowl) in the combined aa sequences of the seven conserved domains in the viral replicase gene, namely ADRP in nsp3, nsp5, nsp12, nsp13, nsp14, nsp15, and nsp16, is 85.2–88.8% with the mean of 86.4% ± 1.1%. In contrast, the corresponding aa identity is 94.5–99.2% with the mean of 96.0% ± 0.9% among the 11 CdCoVs, and 94.5–99.2% with the mean of 96.0% ± 2.5% among the three DdCoVs. Therefore, according to the sole species demarcation criterion for CoVs that the viruses sharing more than 90% aa sequence identity in these seven conserved domains belong to the same species [8], the three DdCoVs and the 11 CdCoVs should belong to two different species.

**Fig 3:**
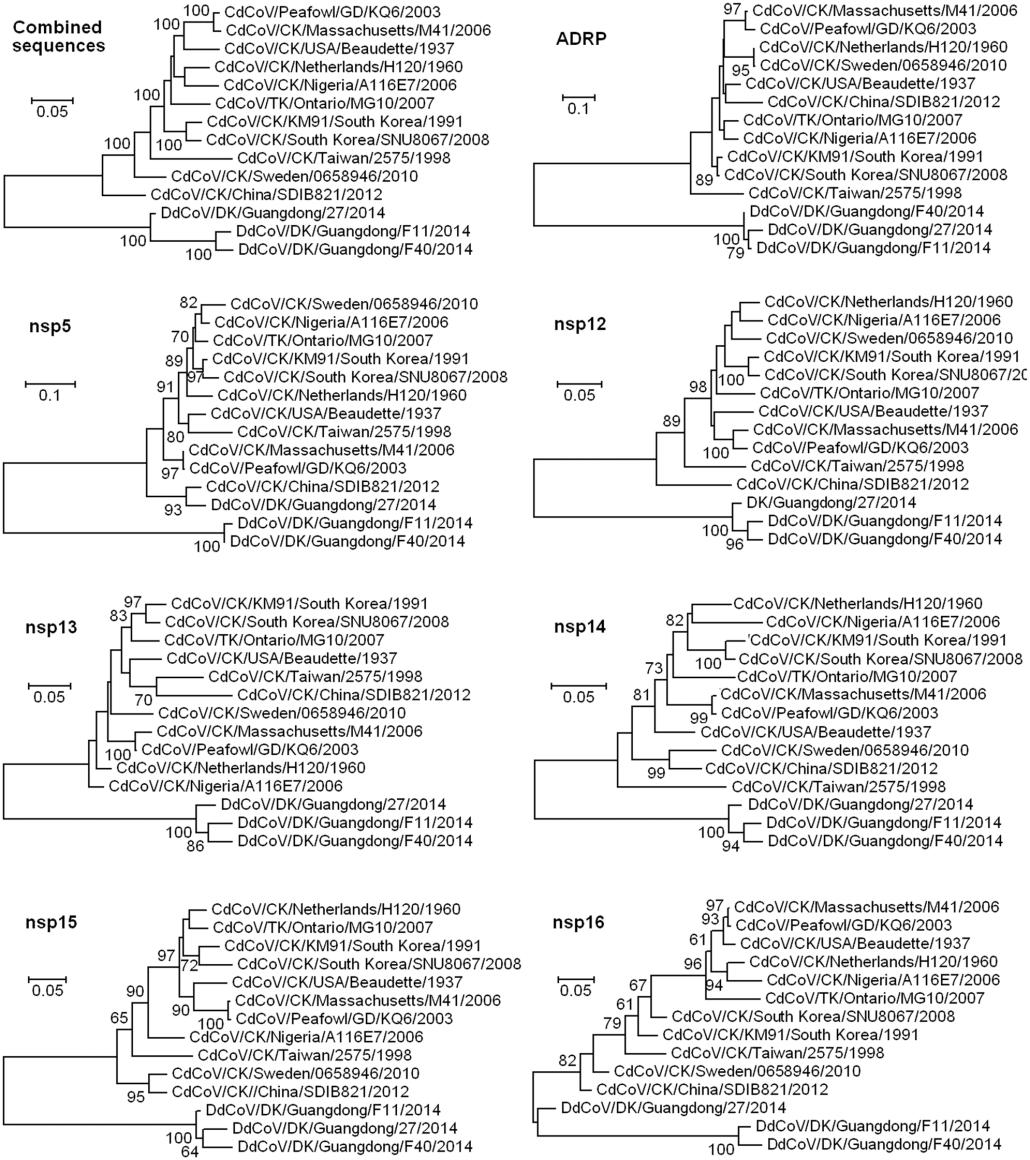
Phylogenetic relationships of 12 CoVs based on the sequences of seven conserved domains in their genomes. The first tree was based on the sequences of the seven domains combined together, and the remaining trees were based on the sequences of each of the seven domains.

Phylogentic analysis of the sequences of the seven domains combined together, as showed in Fig 3, suggested that the three DdCoVs and the 11 CdCoVs belong to different lineages, supporting that they belong to two different species. The three DdCoVs and the 11 CdCoVs also belong to different lineages based on each of these seven conserved domains with the exceptions that DdCoV/GD/2014 is located in the same lineage with the 11 CdCoVs based on the nsp5 gene sequences and the nsp16 gene sequences (Fig 3). These two exceptions suggested that the two genes of DdCoV/GD/2014 likely originated from CdCoVs through genomic recombination.

### Recombination analysis

As showed in Table 1, we identified two potential recombination events with high confidence between the lineages of CdCoV and DdCoV using the software RDP, based on the combined sequences of the seven conserved domains of the aforementioned three DdCoVs and the 11 CdCoVs. These two potential recombination events were the same as suggested through the phylogenetic analysis described above. Therefore, our phylogenetic and recombination analysis both suggested that genomic recombination has likely occurred between the lineages of CdCoV and DdCoV.

**Table 1:** The potential recombination events between the lineages of CdCoV and DdCoV identified using the tool RDP.

The recombinant	The parent	Recombination region	Nucleotides ^a^
DdCoV/GD/2014	The CdCoV lineage	The major part of nsp5	23–920
DdCoV/GD/2014	The CdCoV lineage	The major part of nsp16	65–695

^a^ The nucleotides involved in the recombination were numbered from the first coding nucleotide of the genes.

### Surveillance of CoVs in poultry and phylogenetic analysis of the conserved RT-PCR amplicons

**Fig 4:**
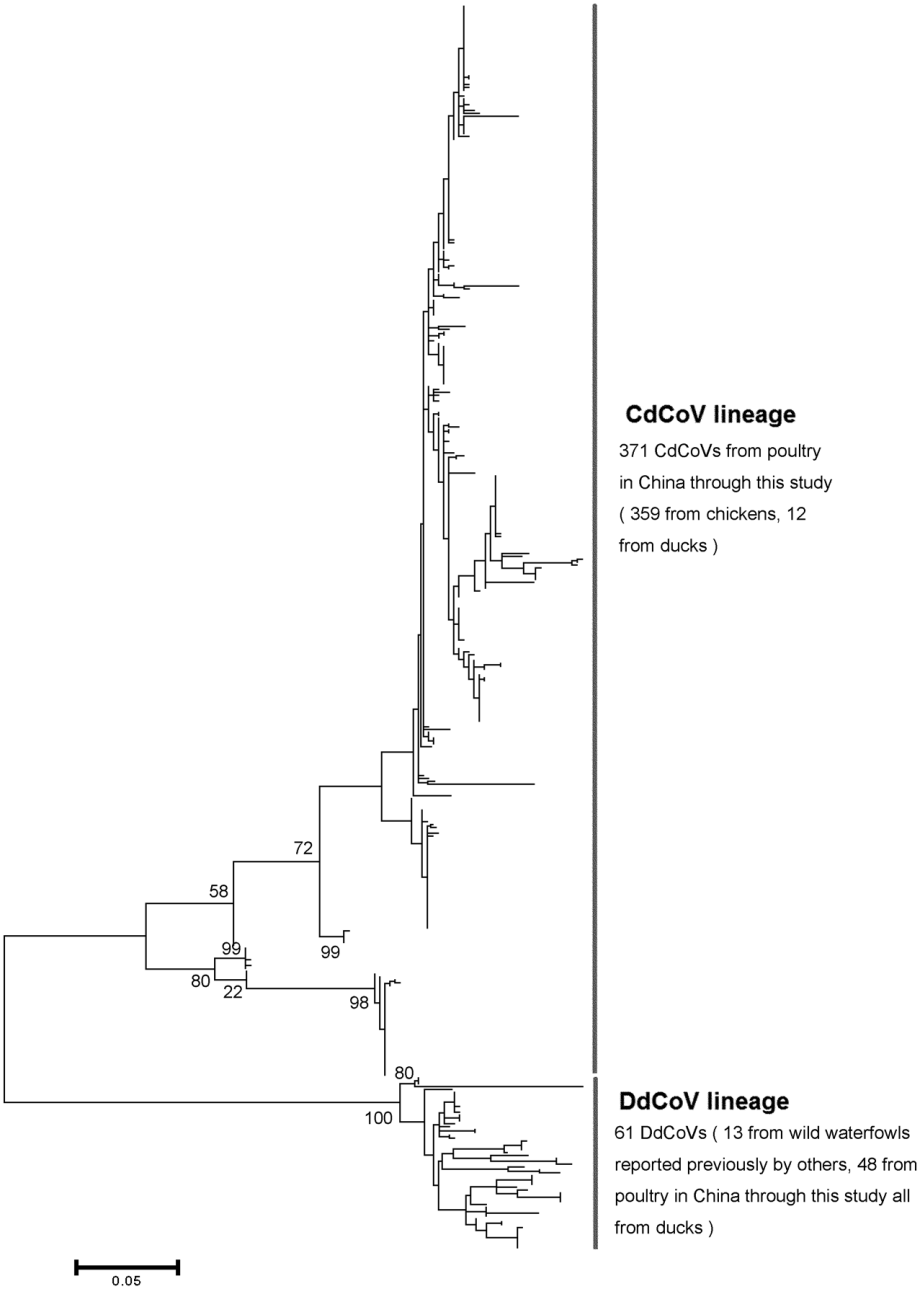
Phylogenetic relationship among the CoVs identified through a large-scale surveillance based on the sequences in the replicase gene amplified by the conserved RT-PCR.

A total of 419 CoVs were identified from the 3467 swab samples detected through inoculation in embyonated eggs followed by the conserved RT-PCR assay and the 116 feces or drinking-water samples detected directly using the conserved RT-PCR assay. Phylogentic analysis of the RT-PCR amplicons suggested that these 419 viruses could be classified into two lineages corresponding to CdCoVs (*n* = 371) and DdCoVs (*n* = 48), respectively, as showed in Fig 4.

**Fig 5:**
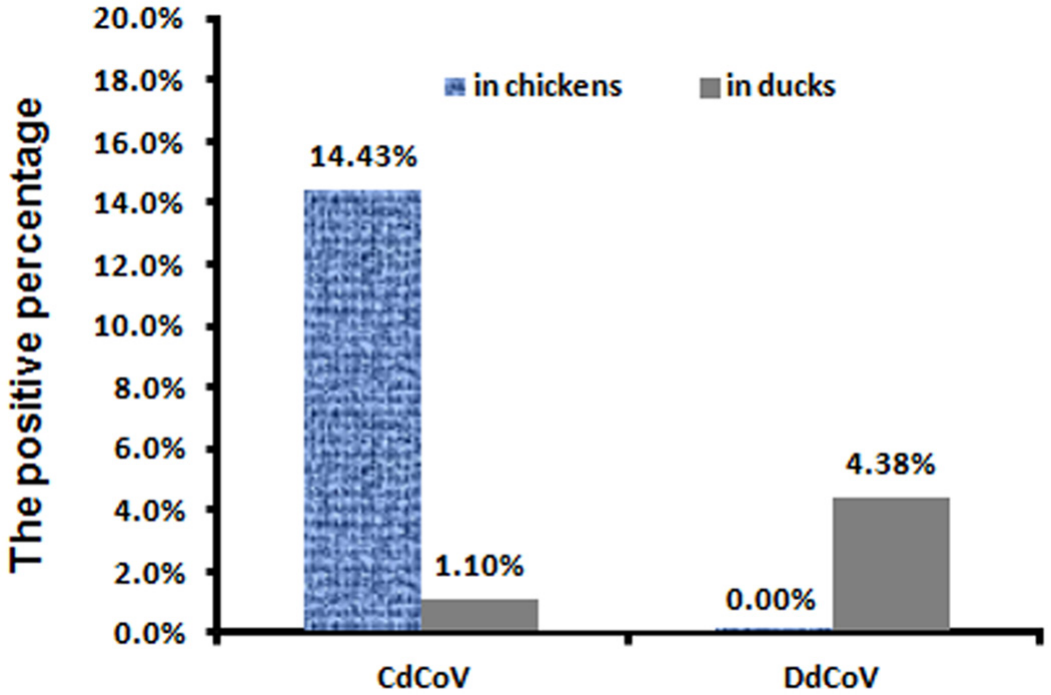
The positive percentage of CdCoV and DdCoV in chicken and duck samples.

As showed in Fig 5, the positive percentage of CdCoVs in the samples of chickens, 14.43% (359/2488), was significantly higher than that in the samples of ducks, 1.10% (12/1095), with *P* < 0.01 by the Chi-square test. The positive percentage of DdCoVs in the samples of ducks, 4.38% (48/1095), was significantly higher than that in the samples of chickens, 0.00% (0/2488), with *P* < 0.01 by the Chi-square test. Therefore, the data confirmed that CdCoVs and DdCoVs mainly circulate in chickens and ducks, respectively, although CdCoVs can circulate in ducks at a relatively low prevalence.

CdCoVs were found in 80.0% (4/5) of the provinces, 82.14% (23/28) of the LPMs, and none of the 14 duck farms or the two of the backyard flocks, where the samples were collected, and DdCoVs were found in 80.0% (4/5) of the provinces, 46.43% (13/28) of the LPMs, 7.14% (1/14) of the 14 duck farms, and none of the backyard flocks where the duck samples were collected. These data suggested that both CdCoVs and DdCoVs are prevalent in LPMs in some regions in China.

Of the aforementioned 48 DdCoVs from duck samples, seven were from duck swab samples, 29 from duck feces samples, and 12 from duck drinking-water samples. The prevalence of DdCoVs in duck swab, feces, and drinking-water samples were 0.7% (7/982), 41.43% (29/70), and 27.9% (12/43). All the collected duck feces and drinking-water samples were found to be CoV negative when they were detected through inoculation in chicken embyonated eggs followed by the conserved RT-PCR assay. This indicates that DdCoVs replicate poorly in chicken embyonated eggs, as we reported previously [12].

## Discussion

Unlike cellular organisms including prokaryotes and eukaryotes, viruses usually harbor no repeats in their genomes. Therefore, it is surprising to identify a tandem repeat in the genomes of DdCoVs in this study. To our knowledge, of all known vertebrate RNA viruses, tandem repeats have been identified only in the genomes of some human HKU1 CoVs which contain 2 to 17 copies of a 10-aa peptide (NDDEDVVTGD) with unknown origin and functions [7]. The tandem repeats in both DdCoVs and HKU1 CoVs are located at highly variable regions in the PL1^pro^ domain of nsp3. Because nsp3 is not essential for the virus replicating in cultured cells [8], and tandem repeats do not exist in many other CoVs, these tandem repeats may be of little importance for the virus replicating in cultured cells. On the other hand, as tandem repeats have not been identified in any known vertebrate RNA viruses except in two species of CoVs, CoVs might have an intrinsic inclination to generate tandem repeats.

CoVs mutate fast due to their frequent genomic recombination and the limited proofreading capacity of their RNA replicases [8, 29]. We demonstrated here that, as compared with CdCoV, DdCoV has accumulated many substitutions and indels in its genome, and recombination events have occurred between CdCoV and DdCoV. Moreover, quite a few indels resulted in frameshifts in the viral genomes, which are rare in other viruses as frameshifts usually change greatly the aa sequence of a gene.

This study showed that the genetic distances between DdCoV and CdCoV are large enough to separate them into different species in the genus *Gammacoronavirus*, according to the official criteria of ICTV [8]. The large-scale surveillance we reported here confirmed that DdCoV and CdCoV mainly circulate in ducks and chickens, respectively, and they belong to different phylogenetic lineages. Therefore, classifying DdCoV and CdCoV into different species also meets the definition of virus species that a virus species is a polythetic class of viruses that constitutes a replicating lineage and occupies a particular ecological niche [8].

Currently, *Avian coronavirus* is the designation of the species within *Gammacoronavirus* covering IBVs in chickens and related CoVs in turkeys and other birds. The designation has become questionable because some CoVs in wild birds have been identified to be members of another genus *Deltacoronavirus* [9]. In this report, we further challenged the designation of *Avian coronavirus* as we provided solid evidences in supporting that the CoVs dominant respectively in ducks and chickens likely belong to different species. These two potential species can be designated as *Chicken-dominant coronavirus* and *Duck-dominant coronavirus*, and the designations allow that these CoVs may also circulate in turkeys, geese, peafowl, or other birds at a relatively low prevalence.

Fig 1 suggested that some CoVs from wild migratory waterfowls reported previously by others may belong to the same species with DdCoVs [8, 12, 16–18], indicating that DdCoVs may widely distributed not only in domestic ducks, but also in wild migratory waterfowls.

Our surveillance results indicate that DdCoVs likely replicate poorly in chicken embyonated eggs. Therefore, the detection procedure with inoculation in chicken embyonated eggs is not suitable for the detection of DdCoVs, although it is suitable for surveillance of avian influenza viruses, Newcastle viruses, and CdCoVs. Consequently, the prevalence of DdCoVs in duck swab samples detected with inoculation in chicken embyonated eggs could be greatly underestimated in this study.

Our surveillance results suggest that CdCoVs and DdCoVs are of high prevalence at LPMs in some regions in China. Therefore, LPMs likely play an important role in the circulation of CoVs in poultry, as they do in the circulation of avian influenza viruses [30].

In conclusion, this study shed novel insight into the genetic diversity, distribution, evolution, and taxonomy of the coronaviruses in poultry.

## Supporting Information

S1 Table The primers used for amplification and sequencing of the genome of DdCoV^a^. (DOCX)

S1 Fig The indels in the aligned sequences of the 1ab genes of two CdCoVs and one DdCoV which terminated at sites 20006, 20079, and 20076, respectively. The deleted nucleotides at the indel gaps were showed with “-”. Many sites with no gaps were replaced with the marks of rectangle blocks. The sites without substitutions among the viruses are marked with asterisks. The sites with frameshifts were indicated with red numbers.
(TIF)
