# Supplementary material for: Genomic Analysis and Surveillance of the Coronavirus Dominant in Ducks in China
Source: PLoS One. 2015 Jun 8;10(6):e0129256. doi: 10.1371/journal.pone.0129256 (PMC4459809; doi:10.1371/journal.pone.0129256)
Supplement: S1 Table — (DOCX) [file pone.0129256.s001.docx]

**Table S1.** The primers used for amplification of the genome of DdCoV ^a^.

| Pair | Amplified region ^b^ | Forward primer sequence | Reverse primer sequence |
| --- | --- | --- | --- |
| 1 | 175–1230 | TGTTGCTGGTATCACTGCTTG | AGTAATTGACCTAGCACACTTGGCAAA |
| 2 | 808–1734 | TTGGCAGATTTAGAAGACATCTTTGG | TTTAAGAGCTGTTACAAGCTCAGAGACA |
| 3 | 1579–3818 | GCACCTCTTTCAGGAAATTTTG | ACATTATTCACACATTGAATGCC |
| 4 | 2228–3725 | AAAAGTGTGCCATAGGTAGAAT | ACAAACTTATTACCACAAAAGTCTGC |
| 5 | 2516–3725 | TTGGTGCAGTTAATGTTGTTTG | ACAAACTTATTACCACAAAAGTCTGC |
| 6 | 2228–3878 | AAAAGTGTGCCATAGGTAGAAT | TTCTTGTATGCAGCAACAAGC |
| 7 | 2516–3878 | TTGGTGCAGTTAATGTTGTTTG | TTCTTGTATGCAGCAACAAGC |
| 8 | 3641–4101 | GCATTGTAAATGCTGCWAATGA | GGAGCGGTATTTAACACCATC |
| 9 | 4072–5934 | CTTACGGAGGATGGTGTTAAWTAC | ACAATATCGTAACACATCAAAA |
| 10 | 5458–6865 | TGGCGTGCAGAACACCTTAATA | CACTCTTAGACACAGGCTCTACTAC |
| 11 | 6423–7517 | TTGGTATTGTAGAAATTGTGATG | CTAAGAACACCTTTATCTATAAC |
| 12 | 6841–8292 | GTAGTAGAGCCTGTGTCTAAGAGTG | AGCTTTAAAAACACCTTGAAACTT |
| 13 | 7495–8732 | GTTATAGATAAAGGTGTTCTTAG | CCTGAATAGTACTTTAATCTAGCATAC |
| 14 | 8023–8966 | TATTGTAGGGGTAGTGTGTGTGAG | TCACCTAGCCACAGTCCATTAAG |
| 15 | 8706–9746 | GTATGCTAGATTAAAGTACTATTCAGG | GGTGTCAATTCATCTTCAAAAT |
| 16 | 9614–11397 | CCGCTATTACTAAATTAAGTGCTATAAC | GTCTATATTCCAAACAACTGTTGA |
| 17 | 11245–12403 | TTTGACCAGGCTAGTAGTGGTGTTGT | TTTCCGTCAGACAGGGGTATCA |
| 18 | 12172–13779 | GGTTCTTTTGTKCAAATACCTACT | CAAAGTATTTAGAAGTCAC |
| 19 | 13664–14563 | TCCTCAAACTGGTAATGCTGCTA | TCAACAAAGGCTGGGTCAAAA |
| 20 | 14112–15682 | AAGTTTTATGGCGGTTGGGA | CCTGTGAAAACATAATTTCTAT |
| 21 | 15661–18250 | ATAGAAATTATGTTTTCACAGG | AAAAATGGCATAGCTTTCAAA |
| 22 | 17801–20291 | GGATTTGACATACCCTCACATTG | TGGCGTTGCTTTCAATCTCA |
| 23 | 19528–20268 | ATAATTTGCCTGAACTTTATAAAGT | CAAACTTAGCAACGTCAAATATACTA |
| 24 | 20188–21192 | TGCACGCAAATTATATATTTTGGA | CCGAAAAATGAACTAGAGCATTA |
| 25 | 20188–23514 | TGCACGCAAATTATATATTTTGGA | TTTGTCAAAAAGwARGTCTTCAAT |
| 26 | 23065–23733 | CAGAAAGTAGAGGAGTGTGTTAAGT | ACCACAACAACCAGTCATGAA |
| 27 | 23713–25995 | TTCATGACTGGTTGTTGTGGT | GGCTTTTATTGCTTGAAACCAAGA |
| 28 | 25258–26168 | TTTGAGGATACTAATATAAATCCTCTT | CCTGTGCCAGTGTAATAAAAGTACCA |
| 29 | 26140–26646 | GCGTGGTACTTTTATTACACTGGC | CATCTTGTCATCACCGAAGTTGC |
| 30 | 26272–27570 | CCTGATAAGTTCGACCAATACC | CTTAGCCAATTAAACTTAACTTAAAC |
| 31 | 27343–Poly(A) | TGCCTATCGCCAGGGAAATGTCTAAT | GGGCGGTTTTTTTTTTTTTTTTTTTT |

^a^ The pairs 4–6 were also used for amplification and sequencing of the region covering a tandem repeat are showed in shadow.

^b^ The nucleotides were numbered from the first coding nucleotide of the 1a gene after the partially sequenced 5′-NCR.
