# Supplementary figures and images for: Genomic Analysis and Surveillance of the Coronavirus Dominant in Ducks in China
Source: PLoS One. 2015 Jun 8;10(6):e0129256. doi: 10.1371/journal.pone.0129256 (PMC4459809; doi:10.1371/journal.pone.0129256)

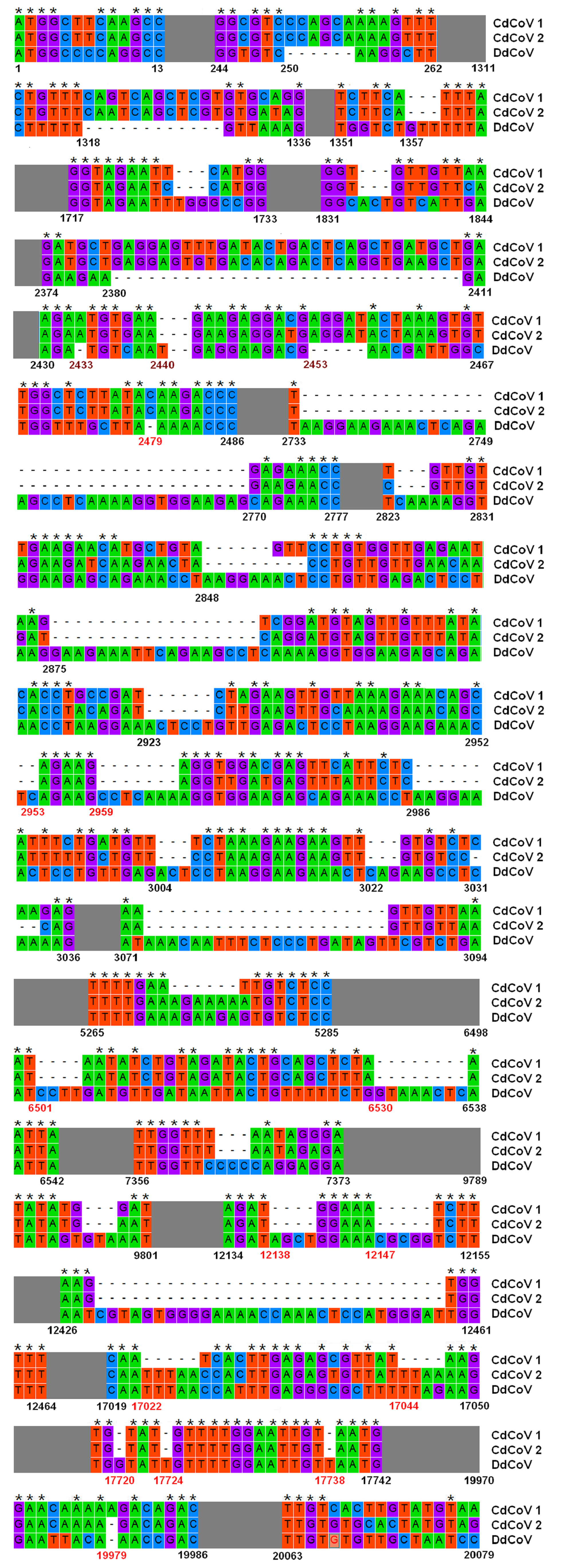

Supplement: S1 Fig — The deleted nucleotides at the indel gaps were showed with “-”. Many sites with no gaps were replaced with the marks of rectangle blocks. The sites without substitutions among the viruses are marked with asterisks. The sites with frameshifts were indicated with red numbers. (TIF) [file pone.0129256.s002.tif]
